# Rural work and COVID-19: challenges and proposals for the prevention of damages

**DOI:** 10.47626/1679-4435-2021-742

**Published:** 2023-08-08

**Authors:** Lorhany dos Santos Santana, Magno Conceição das Merces, Thais Regis Aranha-Rossi, Antonio Marcos Tosoli Gomes, Jeane Freitas de Oliveira, Paulo José Bastos Barbosa, Amália Ivine Costa Santana, Marcio Costa de Souza, Anderson Reis de Sousa

**Affiliations:** 1 Departamento de Ciências da Vida, Universidade do Estado da Bahia (UNEB), Salvador, BA, Brazil; 2 Faculdade de Enfermagem, Universidade do Estado do Rio de Janeiro (UERJ), Rio de Janeiro, RJ, Brazil; 3 Escola de Enfermagem, Universidade Federal da Bahia (UFBA), Salvador, BA, Brazil; 4 Hospital Universitário Professor Edgard Santos, UFBA, Salvador, BA, Brazil

**Keywords:** pandemics, coronavirus infections, occupational health, COVID-19, pandemias, infecções por coronavírus, saúde do trabalhador, COVID-19

## Abstract

The COVID-19 pandemic has highlighted the inequalities in health care access in Brazil, exarcerbating vulnerabilities and social determinants of health. Inequality is part of the context of rural populations, especially rural workers and family and subsistence farmers due to both the direct consequences of work activity and the unfavorable socioeconomic context, especially regarding service provision, the guaranteeing of rights, and the coordination of care networks. This article reviews challenges to the health of these workers and outlines proposals for disease prevention in the context of the COVID-19 pandemic.

## Abstract (pt)

Apandemia de covid-19 no mundo trouxe à tona as desigualdades vivenciadas no acesso à saúde no Brasil, escancarando as vulnerabilidades e os determinantes sociais de saúde. É nesse ínterim de iniquidades que as populações rurais se inserem, em especial trabalhadores e trabalhadoras rurais e membros da agricultura familiar e de subsistência, tanto pelas consequências diretas da atividade laboral quanto pelo contexto socioeconômico desfavorável, especialmente no que diz respeito a oferta de serviços, garantia de direitos e articulação das redes de assistência. Frente a isso, o presente trabalho discute os desafios relacionados à saúde desses trabalhadores e traça propostas para a prevenção de agravos da referida população no contexto da pandemia de covid-19.

## INTRODUCTION

In December 2019, pneumonia cases of unknown origin began appearing in Wuhan, China. The etiological agent was later identified as severe acute respiratory syndrome coronavirus 2, a subtype of the *Coronaviridae* family known to cause respiratory infections in humans, and it was designated coronavirus disease 2019 (COVID-19). In March 2020, the World Health Organization raised COVID-19 to pandemic status, declaring it a public health situation of international importance. Collective efforts began worldwide to better understand the new disease and devise strategies to contain its spread.^1^

It became clear that COVID-19’s morbidity and mortality rates were higher among older adults and those with comorbidities, such as hypertension, diabetes, heart disease, as well as among those with underlying respiratory diseases, such as asthma and chronic obstructive pulmonary disease. The disease also spread faster and with higher risk in regions with unfavorable socioeconomic and demographic indicators.^2^

Rural Brazil is one such vulnerable population, having higher illiteracy and lower education than urban residents, in addition to lower income and human development index.^2^ Service distribution is another factor due to less complex health care networks, greater difficulty of access, and fewer professionals.^2^ These regions also face other challenges, such as traditional practices, labor relations, and gender roles.^2^

The way that work is configured as a social determinant of health depends on several factors involved in the social division of labor. In *The Principles of Scientific Management,* Frederick Winslow Taylor investigated how the hierarchical management of work tasks could result in greater productivity.^3^ Likewise, gender roles affect the assignment and performance of work processes.^4^ However, such divisions are not restricted to work environments, and vulnerabilities become even more intense when other risk factors in the sociocultural and economic spheres are considered, intersecting in the lives of workers.

Rural work is largely agricultural in nature, and agriculture is one of the main sources of income in the Brazilian economy. In 2015, agriculture represented 4.46% of the gross domestic product, being third largest sector, behind only the service and industry.^5^ Although practices that have come to be called “agribusiness” would seem to promote production, it must be pointed out that, in addition to driving environmental imbalance, they intensify socioeconomic inequality, eradicating small producers, family and subsistence farms, and rural workers from the social structure.^6^ However, these effects are neither coincidental nor unnoticed, since they also erode sociopolitical groups, weakening their representation in political-economic processes. Thus, the base economy becomes less diverse and the rights of the rural population are less defended, including their social security, safety, and health, as well as access to basic services, especially health and education.^7^

Several studies have shown that rural workers have a higher prevalence of respiratory symptoms and higher morbidity and mortality rates due to respiratory problems, stemming from continuous exposure to dust, pesticides and chemical residues, in addition to socioeconomic variables and lifestyle habits, such as the use of cigarettes, snuff, and wood stoves.^8,9,10^

Historically, Brazilian social and political movements have advocated for improved living and working conditions for rural workers. However, their situation is characterized by numerous overlapping labor and health vulnerabilities, precarious legal protection, a lack of access to personal and collective protective equipment, and demeaning conditions, resulting in a higher risk of COVID-19 contamination and greater severity of infection. Thus, we aim to discuss proposals for and challenges to preventing health problems among rural workers in the context of the COVID-19 pandemic in Brazil.

## CHALLENGES FACED BY RURAL WORKERS IN THE CONTEXT OF COVID-19

The health of rural populations in Brazil is vulnerable on a number of fronts, especially social and economic ones. Many remote locations are beyond the reach of Internet and cellular service networks, thus barring access to telemedicine or guidance about protective measures against COVID-19.^11^

Health care networks in rural areas are less structured (eg, no intensive care units, mechanical ventilators, defibrillators, or similar devices), are less complex, and are unevenly distributed. Thus, in many areas, health professionals, especially physicians, are lacking and specialized centers for more serious cases are hundreds of kilometers away. However, in the rare situations where mobile emergency services are available, adequate professional follow-up is still lacking, representing a further challenge.^12^

The rate of COVID-19 testing per million inhabitants is also lower in rural areas than the national average. In the USA, it is approximately 27% lower, including underreporting and underestimation of the number of cases.^12^ Thus, although lower population density is a protective factor against the pandemic,^12^ the epidemiological profile of rural populations increases the risk of death by COVID-19, and this profile is aggravated by infectious-parasitic diseases and other factors associated with vulnerability, such as alcoholism, violence, and mental disorders.^13^ Rural areas also have a large number of older adults and people with comorbidities, known risk factors for COVID-19. Thus rural cases tend to be more serious, especially considering the greater precariousness of local health care.^12^

Another challenge is the displacement of people from urban to peri-urban and rural areas. With the interruption of commercial and educational activities during the pandemic, people migrated to less populated areas for social isolation and quarantining. In India, for example, the government initially encouraged migration to rural areas but then recanted when statistical data revealed the danger of displacement: viral dissemination occurs along transport routes, thus risking the health of rural populations, ie, the disease is taken from large centers, which have larger and more complex health care networks for severe cases, to more remote regions with fewer health professionals and less treatment infrastructure.^11^

In China, it is estimated that between 30 and 50 million migrant workers lost their jobs by the end of March 2020 due to the pandemic, which exacerbated preexisting inequalities. Rural migrant workers had more difficulty obtaining employment than urban workers, ie, the less educated and less qualified, the higher the unemployment rate.^14^

Many rural communities also have unique cultural characteristics. Religious rituals, spiritual activities, and the use of plants and objects in treatment apart from scientific evidence are not uncommon and must be treated ethically and respectfully by local health authorities to avoid alterity in dialogue with the population and inefficiency in fulfilling standard recommendations and guidelines. Effective communication of health policies must be maintained to prevent even greater risk in this population during the pandemic.

Another aggravating factor is that the quarantine did not interrupt rural work. Labor relations in this context are characterized by a lack of formal documented employment that would ensure minimum employment conditions, ie, contracts are usually verbal, pay is very low, and little effort is made toward work safety. In 2012, a survey conducted by the Interunion Department of Statistics and Socioeconomic Studies (DIEESE),^15^ based on Brazilian Institute of Geography and Statistics (IBGE) data, found that approximately 60% of salaried rural workers are employed informally, totaling approximately 2.4 million people (1 million of whom are in the northeast region). In the state of Bahia, the informal employment rate is 81.7%.

Quite often, in addition to extreme sun exposure, these workers use improvised personal protective equipment, especially for the respiratory-tract, to avoid inhaling organic residues, dust, and other components. In view of their socioeconomic conditions as small producers, family, or subsistence farmers, whether as autonomous or informal employees, necessity demands that they break social isolation. Many of these workers are forced to decide between protection and income.^16^

It should also be considered that a large portion of this population is older adults who have already retired but continue routine activities in the field, either out of habit or necessity. Although this situation would exist regardless of the pandemic, it becomes an aggravating factor given current conditions.

The behavioral factor of resistance should also be discussed, since many question whether the virus, the pandemic, or the data reported in the media are actually real. This indirectly demonstrates that information strategies and guidelines are not as effective as health authorities would like to believe. Thus, other forms of health education intervention should be considered for rural populations to reduce infection and transmission.

## STRATEGIES TO PREVENT INFECTION AMONG RURAL WORKERS

Regarding COVID-19 prevention strategies, the Ministry of Health points out that no single rule can be applied to the entire country. Each region must evaluate, together with local authorities, what should be done on a case-by-case basis, bearing in mind the complex geography of Brazil, the different social contexts and, above all, health care inequality. This includes case and risk scenarios, since different states have not had the same experiences.^17^

Considering that approximately 80% of COVID-19 cases only involve mild symptoms and 20% require care from the hospital network, the need for primary health to cope with the ongoing pandemic is clear. Care must be provided for confirmed or suspected cases based on adequate clinical testing, in addition to medication for symptomatic cases and monitoring/telemonitoring for early warning signs among those who may require hospital care.

In addition to these measures, other fundamental elements in caring for rural workers with COVID-19 include social isolation, tracking, and infection precautions for workers and their contacts, as well as guaranteeing subsistence for their families. Social networks are also important for highly vulnerable individuals affected by the disease and for those who live in hard to reach areas, eg, transportation, instruction, and help adhering to the sanitary measures recommended by health authorities. Finally, we emphasize the need to organize this line of care in this specific context during the pandemic.

The need for coordination between epidemiological, sanitary, environmental, and labor surveillance agencies in rural settings should also be pointed out, considering that the role of health surveillance is to act continuously and systematically, adapting to the needs of each context regarding injury prevention, health promotion, and risk control.^17^

Thus, in vulnerable groups, eg, rural populations during the COVID-19 pandemic, intersectoral interventions must be coordinated with family health units. It is also essential for health surveillance agencies to align with the National Policy for Comprehensive Health Care for the Population of the Countryside and Forests to strengthen and amplify the care outlined in this public policy.^18^

The planning and implementation of these public health measures must involve active participation from the community so that communication between institutions occurs ethically and efficiently and local needs are addressed. Educational activities, for example, may involve community leaders and include collaborative skills (the professionals, users, and their families) that consider the multidisciplinary character of health practices. It is also of fundamental importance to map areas to which people from large urban centers have migrated in order to conduct health education activities and guarantee the safest and most efficient quarantining possible, including concrete measures such as providing temporary housing.

The federal response to the COVID-19 pandemic in Brazil has been heavily criticized due to the rapid spread of the disease, the defense of “vertical isolation” and the focus on economic activity, in addition to a lack of national coordination and a rapid succession of ministers of health, among other tactics that have contributed to the worsening of the pandemic.^19^ However, the role of the Unified Health System stands out, as does that of the National Health Surveillance System, which having been developed over many years, can investigate, notify, and monitor COVID-19 cases so that strategies can carried out at local and regional levels.^20^ Thus, municipal health managers can coordinate with the state for strategic planning and management.

The Unified Health System must also partner with the Public Prosecutor’s Office, the Ministry of Labor and Employment, and other agencies to guarantee help for vulnerable populations, in this particular case, guaranteeing personal and collective protective equipment for rural workers.

Likewise, many of these workers do not have Internet access (due either to lack of a device or lack of service) and thus face a number of obstacles to accessing federal emergency aid to informal and low-income workers. Hence, this population relies on family and friends as a support network. Coping strategies and better organization by public agencies are needed to guarantee safety in aid requests, transportation (from rural to urban areas for banking services), and minimal waiting to receive payment.

At the same time, testing must be guaranteed for rural workers. Testing is one of the most effective measures worldwide against the pandemic. Hence, the more tests that are performed, and the greater the decentralization of the testing sites, the greater the ability for early diagnosis and isolation of mild cases and their contacts, leading to greater epidemiological control of the situation. Thus, training primary health care professionals to perform tests in rural communities is a valid and effective measure to combat the spread of the virus among vulnerable populations.^20^

Finally, another crucial measure is to give visibility to rural workers and make them seen and heard, since little is said about them in either the mainstream or alternative media, which reinforces inequality of services in an already vulnerable and marginalized population. It should also be pointed out that several social and political movements – including institutionalized ones, such as community associations and rural workers unions – arose from rural communities, whose actions were decisive in expanding public policies to guarantee rights and ensure better individual and collective working conditions.^7,21^ Therefore, partnering with these organizations, in addition to being a way of approaching and decentralizing actions to combat COVID-19, is a way of granting autonomy and power to the involved individuals through the institutions they represent (formally and subjectively) and ensuring the permanence of these groups in a social fabric managed under the rules of biopolitics.^22^

Transposing the political, technical, and operational dimensions of health care in Brazil highlights the need to recognize and value the transcultural dimensions of the most diverse contexts, resulting in health care that is sensitive, integral, and does not disrespect popular regional knowledge, rituals, habits, behaviors, norms, or cultural standards. Thus, in the pandemic context, interventions must not only provide health protection for rural populations, but improve their quality of life and access to goods and services, citizenship, and well-being.

## CONCLUSIONS

In view of the above, considering that rural communities, especially workers, are a risk group and live in a context of health vulnerability, measures to counter the COVID-19 pandemic, especially health surveillance, must be carried out, taking the characteristics of each region into account. Overlapping health risk factors must also be considered, such as sex, region, and economic, racial, and cultural aspects. Therefore, health care strategies for rural Brazilian populations must involve a series of efforts to enable access and guarantee equality, acknowledging the right to health care as a fundamental principle, and these efforts must be intensified during emergency public health situations, such as the COVID-19 pandemic.
